# Risk Factors and Preventive Strategies for Dislocation Following Primary Total Hip Arthroplasty: A Large Single-Surgeon Cohort Study

**DOI:** 10.3390/jcm15166401

**Published:** 2026-08-19

**Authors:** Alikemal Yazıcı, Harun Altınayak

**Affiliations:** 1 Department of Orthopedics and Traumatology, Faculty of Medicine, Samsun University, 55090 Samsun, Türkiye; 2 Department of Orthopedics and Traumatology, Samsun Cıty Hospital, 55080 Samsun, Türkiye; harun240507@gmail.com

**Keywords:** arthroplasty, replacement, hip, femoral offset, femur head, hip dislocation, joint instability, risk factors

## Abstract

**Objective:** This study aimed to analyze the incidence and multifactorial causes of dislocation following primary total hip arthroplasty (THA) and to evaluate current surgical strategies for the prevention and management of this complication. **Methods:** This retrospective study included 1066 hips (median age 57 years; 805 females and 261 males) that underwent primary THA performed by a single surgeon between 1996 and 2025. The median follow-up duration of the patients was 46 months (min: 6, max: 357 months), and 68.9% had primary coxarthrosis, while 31.1% had secondary coxarthrosis. In this study, the effects of dislocation causes, age, gender, femoral head size, liner angle, posterior capsular repair, type of anesthesia, fixation method of the prosthesis, etiology, and surgical experience (distribution by years) on dislocation were statistically analyzed. **Results:** The analysis revealed that 44 of the 1066 hips (4.1%) developed a dislocation. The most common causes of dislocation were identified as femoral offset error (40.91%) and acetabular component malposition (31.82%). A statistically significant correlation was found between dislocation development and gender (*p* < 0.001) and femoral head diameter (*p* = 0.006); the dislocation rate was higher in male patients (8.0%) than in female patients (2.9%). A marked reduction in dislocation rates (1.6%) was observed following the routine use of large-diameter femoral heads (32–36 mm). The highest dislocation rate was observed during the early years of surgical experience (1996–2000, 6.8%). No statistically significant differences were found between dislocation development and age (*p* = 0.055), type of anesthesia, type of prosthesis, liner angle, and secondary etiological factors (*p* > 0.05). **Conclusions:** Instability developing after primary THA is a multifactorial complication. In our study, inadequate femoral offset restoration and acetabular component malposition were identified as the most common mechanical causes of dislocation. Male gender was significantly associated with an increased risk of dislocation, whereas the use of larger femoral head diameters was associated with lower dislocation rates. Our findings suggest that meticulous preoperative planning, accurate component positioning, increased surgical expertise, and the use of larger femoral head diameters may play important roles in reducing the risk of instability.

## 1. Introduction

Although advances in surgical techniques for total hip arthroplasty (THA), including minimally invasive approaches and posterior capsular repair, together with improvements in implant technology and design, particularly the use of larger femoral head diameters, have substantially reduced dislocation rates, instability remains a persistent clinical challenge [1,2,3,4,5,6,7,8]. Dislocation following THA is one of the most common early complications and represents the second most frequent indication for revision surgery after aseptic loosening [9,10,11,12,13]. In series published before the widespread adoption of larger femoral head diameters, the reported incidence of dislocation following primary THA ranged from 3% to 17% [4,5,10,11]. In contrast, more recent series reflecting modern implant designs and the routine use of larger femoral head diameters have reported dislocation rates as low as 0.2% to 2% [2,6,9,12,13,14,15]. Nevertheless, instability remains a major concern, particularly in high-risk patient populations and following revision arthroplasty, with reported dislocation rates after revision THA ranging from 8% to 31.7% [2,4,12,13,14,15,16,17].

It has been reported that approximately 60–70% of dislocations occurring after THA take place within the first six weeks postoperatively and most commonly involve the posterior direction. It is reported that posterior dislocations most often occur during a combination of excessive hip flexion, adduction, and internal rotation [7,18,19]. More than 60% of patients who experience a dislocation develop recurrent instability, over half ultimately require revision surgery, and approximately one-third require multiple closed reduction attempts to achieve successful treatment [9,20,21]. These high rates of recurrence and revision underscore the importance of preventing instability, making it one of the most critical determinants of the long-term success of total hip arthroplasty. Instability after primary THA is a multifactorial complication. Among surgery- and implant-related factors, acetabular and femoral component malposition, inadequate restoration of femoral offset, the use of smaller femoral head diameters, soft tissue imbalance, the surgical approach, and surgeon experience have been identified as the principal contributors to instability [2,3,4]. Also, patient-related factors, including neuromuscular disorders, spinopelvic motion abnormalities, a history of lumbar fusion, and poor patient compliance, have also been shown to increase the risk of dislocation [7,8,9,22,23,24]. Late-stage dislocations, on the other hand, are more commonly associated with polyethylene wear, aseptic loosening, and secondary neuromuscular disorders [7,8,9,22,23,24]. Furthermore, demographic characteristics such as advanced age, female gender, obesity, developmental dysplasia of the hip (DDH), and a history of previous hip surgery have also been reported to contribute to the risk of dislocation [18,19,25,26]. Although national arthroplasty registry studies, systematic reviews, and meta-analyses have shown that the use of larger femoral head diameters (32–36 mm) may reduce the risk of dislocation, most of these studies have included heterogeneous patient populations involving multiple surgeons, institutions, and implant preferences. Hence, it is difficult to assess the independent effect of increased surgical experience over time and changes in implant selection on dislocation rates. In particular, longitudinal clinical studies investigating the impact of the evolution of surgical practice over time and the transition to larger femoral heads on instability within long-term primary THA series performed by a single surgeon remain limited.

The aim of this study was to evaluate the incidence of dislocation and the associated risk factors following primary THA, while longitudinally assessing the impact of changes in surgical practice over time, specifically increasing surgical experience and the transition to larger femoral head diameters, on dislocation rates in a consecutive series of primary THAs performed by a single surgeon. Our primary hypothesis was that increasing surgical experience and the widespread adoption of larger femoral head diameters would significantly reduce the rate of dislocation following primary THA.

## 2. Materials and Methods

### 2.1. Participants

**Figure 1:**
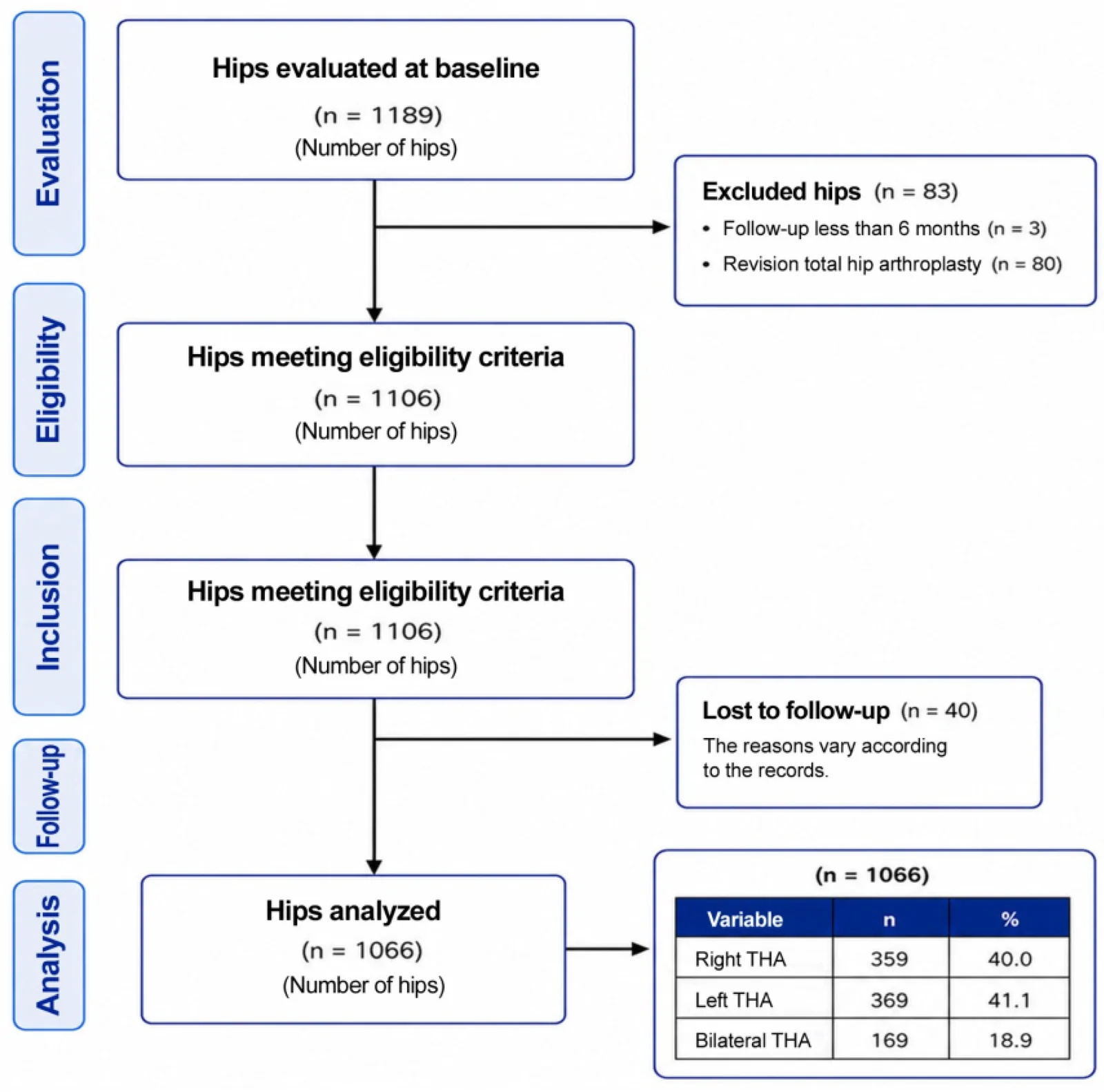
Flowchart.

A total of 1066 hips in 897 patients with a median age of 57 years (range, 21–87 years) who underwent primary THA for primary (68.9%) or secondary (31.1%) hip osteoarthritis were included in this study. A total of 169 patients underwent bilateral primary THA (Figure 1). The study was conducted by retrospectively reviewing the data of patients who underwent THA performed by a single surgeon (AY) between 01 April 1996 and 25 June 2025. Of the patients, 805 (75.5%) were female, and the median follow-up duration was 46 months (minimum: 6, maximum: 357 months [≈29.8 years]). Among secondary hips, 19.61% had DDH, 4.3% had femoral head avascular necrosis (FAVN), 1.41% had rheumatoid arthritis, 0.38% had nonspecific arthritis, 1.31% had ankylosing spondylitis (AS), 1.22% had Perthes sequelae, 1.13% had post-traumatic arthritis, 0.38% had coxarthrosis secondary to collum femoris fracture, 0.66% had coxarthrosis associated with coxa vara, 0.28% had Otto pelvis, 0.38% had coxarthrosis secondary to endoprosthesis, 0.09% had coxarthrosis secondary to femoral head–neck resection, and 0.19% had coxarthrosis following hip osteotomy. Among the cases of DDH, 70.8% were Crowe type I, 10% were Crowe type II, and 19.1% were Crowe types III–IV.

### 2.2. Study Design

This retrospective study, designed to analyze the incidence and causes of dislocation following primary THA and to evaluate preventive strategies, was approved by the Non-Interventional Clinical Research Ethics Committee of Samsun University (Approval Number: GOKAEK 2026/11/7, Date of Approval: 17 June 2026) and conducted in accordance with the principles of the Declaration of Helsinki. Routine clinical and surgical informed consent was obtained from all patients prior to their procedures. The inclusion criteria were defined as undergoing primary THA, having a follow-up period of ≥0.5 years, and the absence of cognitive, linguistic, or mental impairments. The purpose of this criterion is to objectively evaluate surgical and mechanical factors by ruling out dislocations caused by noncompliance with rehabilitation. Primary cases with a follow-up period of less than 0.5 years and cases undergoing revision THA were excluded from the study.

### 2.3. Procedures

Operational data were evaluated by reviewing the retrospective medical records of all participants. According to hospital records, all patients received antibiotic and deep vein thrombosis prophylaxis; 59.4% underwent spinal anesthesia, 27.4% spinal-epidural anesthesia, and 13.2% general anesthesia. It was observed that all hips were operated on using a posterolateral incision [27]. According to implant records, 0.4% of hips underwent cemented THA, 89.8% underwent cementless THA, and 9.8% underwent hybrid THA.

When surgical material details were analyzed, femoral head sizes of 22 mm (2.3%), 26 mm (0.2%), 28 mm (73.9%), 32 mm (10.2%), and 36 mm (13.3%) were used, and 22–26 mm heads were found to be preferentially used in cases of DDH. When implant usage was analyzed by year, it was observed that 28 mm femoral heads were used as standard until 2010, after which 32–36 mm femoral heads were introduced into routine practice. The results showed that 84.5% of the femoral heads used were metal, while 14.6% were ceramic. Also, 0° liners were used in 1.6% of hips, 10° liners in 88.6%, 15° liners in 5.9%, and 20° liners in 5.9%. When capsular management was evaluated, the results indicated that until 2007 only the external rotators were repaired and both the anterior and posterior capsules were excised, whereas from 2007 onwards the posterior capsule was preserved and fixed to the greater trochanter together with the external rotators using No. 1 Ethibond sutures, while only the anterior capsule was excised.

To ensure measurement reliability and maintain data standardization, only hips with 28 mm femoral heads were compared in order to evaluate the effect of posterior capsular repair on dislocation prevention between cases with and without posterior capsular repair. To evaluate the effect of surgical experience on dislocation prevention, dislocation rates over time were compared for periods in which only 28 mm femoral heads were used, stratified by year (1996–2000, 2001–2005, and 2006–2010) following THA. For the clinical evaluation of dislocations, the minimum follow-up period was set at ≥0.5 years. In the systematic evaluation, dislocation occurring within 3 months postoperatively was defined as “early” dislocation, whereas dislocation occurring after 3 months was defined as “late” dislocation. However, no single universally accepted time threshold exists in the literature for defining early and late dislocation; some studies have used 3 months [7,28], 6 months [4], or 1 year [29] as the cutoff point.

The etiology of dislocation was classified primarily on the basis of clinical and intraoperative findings obtained during revision surgery rather than standard radiographic or computed tomography measurements. Inadequate femoral offset restoration was defined by the presence of insufficient abductor soft tissue tension and/or anterior impingement observed during intraoperative assessment of hip motion at revision surgery, followed by restoration of joint stability after increasing the femoral head neck length and/or femoral head diameter to re-establish femoral offset. Acetabular component malposition was defined by intraoperative identification of inadequate acetabular component anteversion and/or inclination during revision surgery, with restoration of stability following repositioning of the component into an appropriate orientation. Accordingly, the etiological classification was based not on objective radiographic measurements but on dynamic intraoperative assessment and the surgeon’s clinical judgment during revision surgery. While this approach more accurately reflects surgical practice, it involves a certain degree of subjectivity because the assessments depend on the observer.

### 2.4. Statistical Analysis

The SPSS program (SPSS for Windows, version 23.0, SPSS Inc., Chicago, IL, USA) was used for statistical analyses. Continuous variables (age and follow-up duration) are presented as mean ± standard deviation (mean ± SD), median (minimum–maximum), and interquartile range (Q1–Q3). Categorical variables are expressed as frequency (n) and percentage (%). The relationships between dislocation and demographic and surgical variables were evaluated using the Pearson chi-square (χ^2^) test for categorical variables. In cases where the expected cell frequencies were less than 5, Fisher’s Exact Test was used instead of the chi-square test, and analyses in which Fisher’s exact test was applied were indicated in the tables with the symbol (a). In the analysis of the age variable, four categories (≤50 years [below middle-age], 51–65 years [middle-age], 66–80 years [young-old], and >81 years [old-old]) determined by the investigators based on the sample distribution and their common use in clinical practice were used. Additionally, an independent samples *t*-test was used to compare continuous variables between cases with and without hip dislocation.

The time to dislocation among the included cases was evaluated using Kaplan–Meier survival analysis. In this method, follow-up duration was defined as the time from the date of enrollment to the detection of dislocation (event) or to the end of the study period (censored data). Cases that did not develop dislocation or whose follow-up could not be completed during the study period were considered censored data and included in the analysis. Cumulative survival rates were updated at each event time and illustrated over time using the Kaplan–Meier survival curve. Mean and median survival times, along with their 95% confidence intervals, were calculated. In cases where the cumulative survival rate did not fall below 50% throughout the study period, the median survival time could not be calculated, and the 75th percentile value was reported instead.

To identify independent risk factors associated with the development of hip prosthesis dislocation, Cox proportional hazards regression analysis was performed. Age, sex, femoral head diameter, hip arthrosis, prosthesis type, capsular repair, and insert angle were initially included in the model. Variables that did not contribute significantly to the model (hip arthrosis, prosthesis type, and capsular repair; *p* > 0.05) were excluded from the final model, and the analysis was repeated including only the statistically significant variables (age, sex, femoral head diameter, and insert angle).

The femoral head diameter variable was initially defined with five categories (22, 26, 28, 32, and 36 mm); however, a model convergence problem was observed due to the extremely low number of cases in some subgroups (particularly 26 mm, n = 2). To resolve this issue and improve statistical reliability, the femoral head diameter variable was recoded into three clinically meaningful categories: 22–26 mm, 28 mm, and 32–36 mm (reference group).

In the final model, age, sex, the recoded femoral head diameter category (reference: 32–36 mm), and insert angle (reference: 20°) were simultaneously entered into the model using the Enter method. The effect of each variable on dislocation risk is expressed as hazard ratio (HR) with 95% confidence interval (CI). The overall significance of the model was assessed using the Omnibus test (χ^2^ statistic).

## 3. Results

Descriptive data are presented in Table 1. A total of 1066 hips in 897 patients with a median age of 57 years (range, 21–87 years) who underwent primary THA for primary (68.9%) or secondary (31.1%) hip osteoarthritis were included in this study. Of the patients, 295 (27.7%) had right hip osteoarthritis, 310 (29.1%) had left hip osteoarthritis, and 461 (43.2%) had bilateral hip osteoarthritis. When analyzed according to the surgical procedure, 359 patients underwent unilateral right primary THA, 369 underwent unilateral left primary THA, and 169 underwent bilateral primary THA. Not all patients with bilateral hip osteoarthritis underwent bilateral arthroplasty; in a subset of these patients, only the symptomatic hip underwent arthroplasty during the study period.

Direction, timing, and annual incidence of dislocations following THA are given in Table 2. Dislocation occurred in 44 of 1066 hips (4.1%). Of the 44 dislocation cases, 43 (97.7%) were posterior, and 32 cases (72.7%) were late dislocations. When the annual distribution of dislocation cases was evaluated, the highest dislocation rate was observed in the 1996–2000 period (6.8%), followed by a gradual decrease in subsequent years. Also, a marked reduction in dislocation rates (1.6%) was observed after the introduction of large femoral head use in 2010.

Data on the causes of dislocations and applied treatment methods are given in Table 3. Based on clinical and intraoperative assessments performed during revision surgery, the most likely mechanisms contributing to the development of dislocation were identified as femoral offset deficiency in 40.91% of cases and acetabular component malposition in 31.82% of cases.

**Figure 2:**
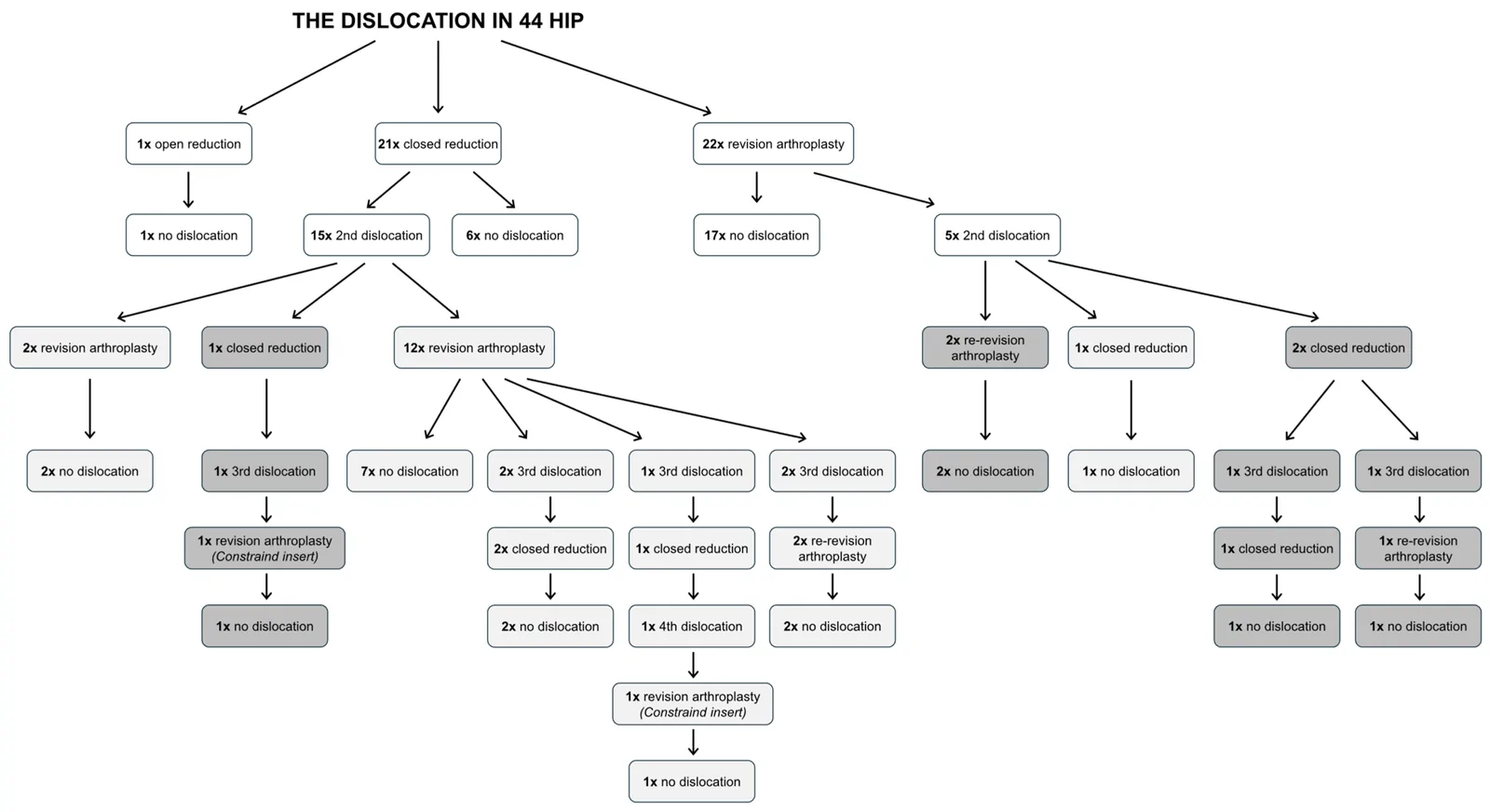
Treatment process for the 44 hips of 42 patients who underwent THA.

The treatment process for the 44 hips of the 42 patients who underwent THA is given in Figure 2. A second dislocation was observed in 20 hips (45.5%). Among cases with a second dislocation, a third dislocation occurred in 6 patients, and among those with a third dislocation, a fourth dislocation occurred in 1 patient. Of the 44 hips, 6 were treated with closed reduction and 1 with open reduction. Revision surgery was performed on 37 hips. During revision surgery, depending on the factors causing dislocation (Table 3), femoral head length and/or diameter was increased, acetabular component revision was performed to correct inclination and/or anteversion, or femoral head revision was additionally performed alongside acetabular component revision. In 2 hips, a constrained liner was used for the treatment of multiple dislocations associated with neuromuscular disease developing after THA and paresis following lumbar spine surgery.

The relationships between dislocation following THA and clinical, demographic, and surgical parameters are given in Table 4. When the age variable was analyzed, the result approached the threshold of statistical significance but did not reach it (χ^2^ = 7.302; *p* = 0.055); however, the highest dislocation rate was observed in patients aged >81 years (7.7%), whereas the lowest rate was observed in the 66–80-year group (1.9%). No statistically significant difference was found in age groups based on hip dislocation status (*p* > 0.05). When analyzed by gender variable, the dislocation rate was found to be significantly higher in male patients (8.0%) compared with female patients (2.9%) (χ^2^ = 13.410, *p* < 0.001). A statistically significant association was found between femoral head diameter and dislocation (χ^2^ = 14.219; *p* = 0.006). Although the highest dislocation rate was observed in the 26 mm femoral head group (50.0%), the very limited number of cases in this group should be taken into consideration. This was followed by the 22 mm (8.0%) and 28 mm (4.7%) groups. However, no dislocations were observed in the 32 mm femoral head group (0.0%), whereas a dislocation rate of 2.8% was recorded in the 36 mm group. No statistically significant differences were observed in the other parameters (*p* > 0.05).

**Figure 3:**
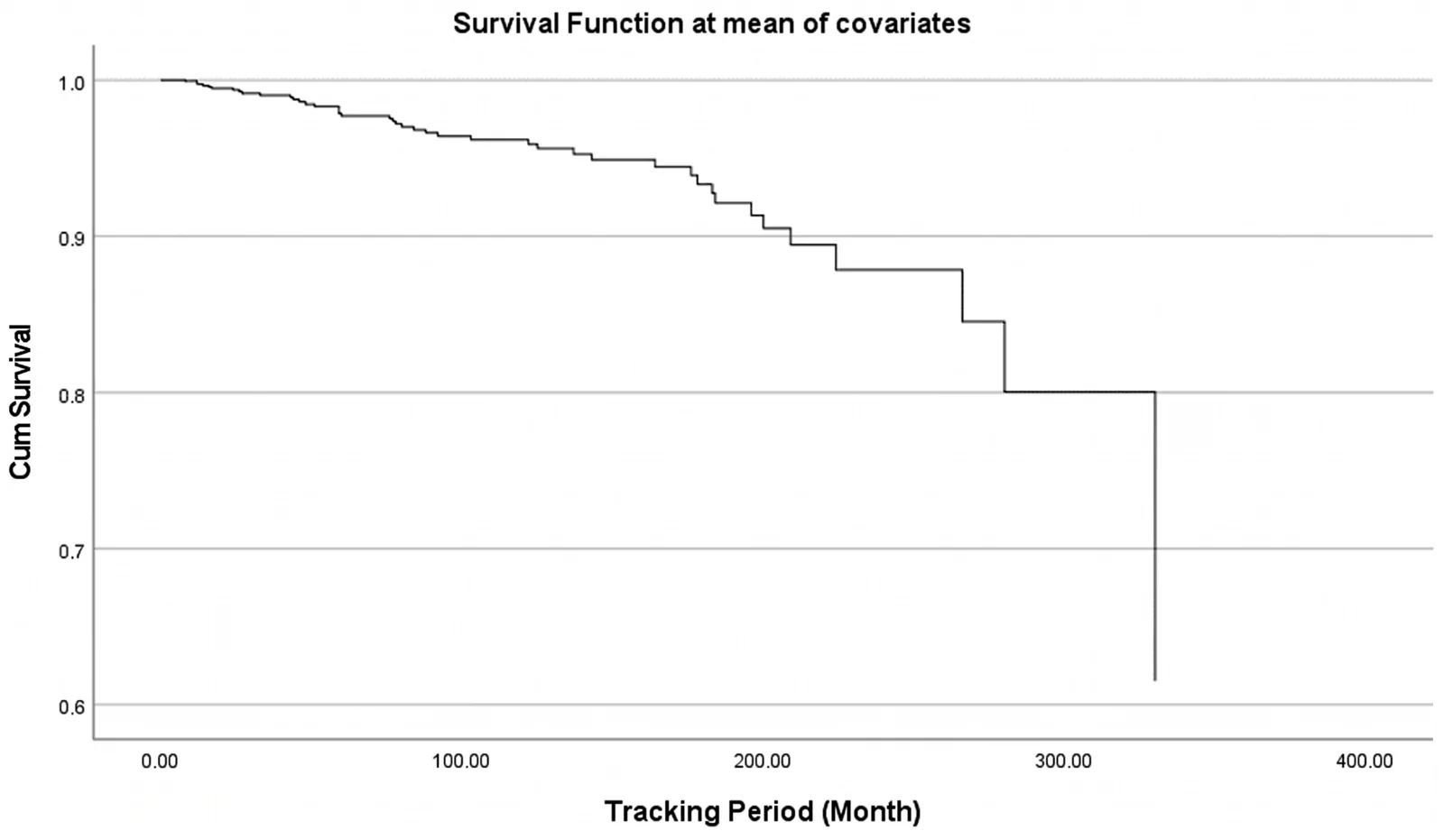
Adjusted survival function showing the cumulative risk of hip prosthesis dislocation based on the Cox proportional hazards regression model.

Cox proportional hazards regression analysis was performed, as shown in Table 5 and Figure 3, to identify independent risk factors associated with the development of hip prosthesis dislocation. The initial model included age, gender, femoral head diameter, hip osteoarthritis type, prosthesis type, capsular repair, and liner orientation. However, hip osteoarthritis type (*p* = 0.235), prosthesis type (*p* = 0.691), and capsular repair (*p* = 0.671) did not make a statistically significant contribution to the model (all *p* > 0.05). Accordingly, these variables were excluded from the model, and the final model, including only the significant predictors (age, gender, femoral head diameter, and liner orientation), is presented in Table 5.

In the initial analysis, femoral head diameter was entered into the model as a five-category variable (22, 26, 28, 32, and 36 mm). However, model convergence problems were encountered because some subgroups, particularly the 26-mm group (n = 2), contained very few hips. Therefore, to increase statistical reliability, the femoral head diameter variable was recoded into three clinically meaningful categories: 22–26 mm, 28 mm (the middle group for comparison with non-reference groups), and 32–36 mm. Following this adjustment, the model was reanalyzed.

A total of 1037 hips were included in the analysis; during the follow-up period, prosthesis dislocation was observed in 44 hips (4.1%), while 993 hips (93.2%) were lost to follow-up. A total of 29 hips (2.7%) were excluded from the analysis because they were censored prior to the first event.

Age, gender, recoded femoral head diameter categories (reference: 32–36 mm), and insert angle (reference: 20°) were simultaneously entered into the model as independent variables using the Enter method. The model was statistically significant (Omnibus test: χ^2^ = 37.762, df = 7, *p* < 0.001).

According to the Cox regression analysis, advanced age (HR: 1.052; 95% CI: 1.022–1.082; *p* = 0.001) and male gender (HR = 3.694; 95% CI = 1.987–6.869; *p* < 0.001) were identified as statistically significant independent risk factors for the development of hip prosthesis dislocation. With respect to femoral head diameter, hips implanted with 22–26 mm femoral heads had an approximately 14-fold higher risk of dislocation than those implanted with 32–36 mm femoral heads (HR = 14.145, 95% CI = 2.647–75.595; *p* = 0.002). With respect to liner orientation, hips with a 0° liner had an approximately 10-fold higher risk of dislocation than those with a 20° liner (HR = 10.233, 95% CI = 1.497–69.951; *p* = 0.018).

Evaluation of dislocation by the etiology of primary and secondary hip osteoarthritis is shown in Table 6. There was no statistically significant difference in the incidence of dislocation between primary and secondary hip osteoarthritis (*p* = 0.870). The underlying causes of secondary hip osteoarthritis are presented only descriptively due to the small number of cases.

**Figure 4:**
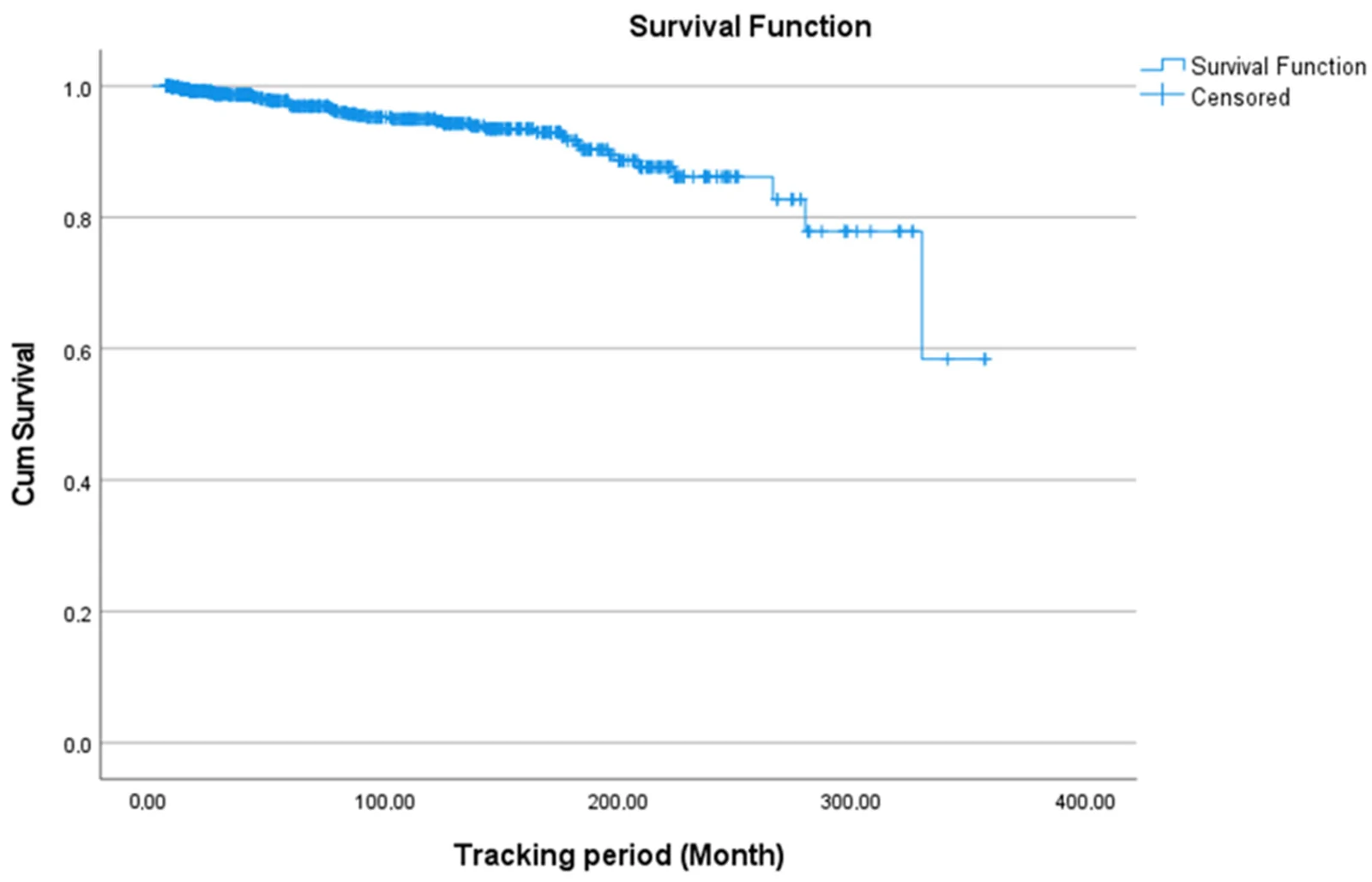
Kaplan–Meier survival curve showing cumulative dislocation-free survival following primary total hip arthroplasty.

In the Kaplan–Meier survival analysis of 1066 hips, 44 hips (4.13%) experienced dislocation during the study period, whereas the remaining 1022 hips (95.87%) were treated as censored observations. The cumulative survival rate was 99.9% at 8 months, when the first event occurred, and declined to 58.4% by the end of follow-up (day 330). The mean survival time was 314.68 ± 7.76 months (95% CI = 299.47–329.89). The median survival time could not be estimated because the cumulative survival probability did not fall below 50% during the study period. The 75th percentile survival time was estimated at 330.00 ± 45.99 months (Figure 4).

## 4. Discussion

The major findings of this study were as follows: among the 44 hips (4.1%) that developed dislocation, the most common presumed cause, based on clinical and intraoperative assessments performed during revision surgery, was inadequate femoral offset restoration (40.91%), followed by acetabular component malposition (31.82%). Following the adoption of 32–36 mm femoral heads, the dislocation rate declined to 1.6%. The highest dislocation rate (6.8%) was observed during 1996–2000, corresponding to the early years of the surgeon’s independent practice, and the dislocation rate was significantly higher in male patients (8.0%) than in female patients (2.9%). Age, type of anesthesia, prosthesis type, and secondary etiological factors (DDH, AS, and FAVN) did not show a statistically significant difference in terms of dislocation development in the overall study population. No statistically significant association was observed between posterior capsular repair and dislocation; however, a clinical trend toward risk reduction was noted. Also, the majority of dislocations were posterior (97.7%) and late in onset (72.7%), and recurrence of instability was observed in 45.5% of the dislocated cases. The study reveals that the instability process after TKA progresses with mechanical-surgical parameters along with demographic or etiological variables, and that the use of a large femoral head combined with increased surgical experience is the most sensitive protective factor in preventing this complication.

In the literature, malposition of the acetabular component is reported as the most common cause of dislocation following THA [8,9], while inadequate restoration of femoral offset is reported as the second most common cause [7,8]. In our study, based on clinical and intraoperative assessments performed during revision surgery, femoral offset deficiency was identified as one of the most commonly observed potential mechanisms. The higher dislocation rate observed in male patients may be attributable to inadequate restoration of the anatomically greater femoral offset during surgery. However, because radiographic measurements of the native and reconstructed femoral offset were not available in our study, this explanation should be regarded as a hypothesis based on our observations rather than as a proven mechanism. Further studies incorporating radiographic measurements of femoral offset stratified by sex are warranted to confirm this association. One of the main causes of dislocation following THA is insufficient femoral offset leading to reduced hip abductor muscle tension, along with a shortened femoral neck. This condition causes the femur to impinge against the anterior rim of the acetabular component, the acetabulum, and the pubis during flexion, adduction, and internal rotation of the hip, resulting in a hinge mechanism. Anterior osteophytes further exacerbate this condition [4,8,9]. In our study, most dislocations were found to occur through a similar mechanism, and in one patient, an additional anterior osteophyte was identified as a triggering factor for dislocation. It has been shown that proper restoration of femoral offset reduces the risk of dislocation following THA [30]. In THA operations, femoral offset has been reported to be associated with abductor muscle function, restoration of head–neck offset, lower extremity length equality, wear, impingement, and dislocation [31].

On the other hand, in our study, acetabular component position error was identified as the second possible cause (31.82%). Lewinnek et al., (1978) [32] reported that the classic safe zone for optimal acetabular component placement is 40° ± 10° for inclination and 15° ± 10° for anteversion. However, in subsequent studies, Dorr et al. (2009) [33] reported that a combined anteversion range of 25–50° (acetabular component plus femoral component) represents a safe zone. However, due to the persistence of dislocations, many authors have stated that it is not possible to define a universal safe zone for component positioning [6]. The authors concluded that acetabular cup position is influenced by multiple factors affecting stability and may vary from patient to patient [34,35,36]. In their study, Ranawac and Maynard [37] demonstrated that, compared with a control group with 47.8° anteversion, both high (72.2°) and low (27.4°) anteversion combinations were associated with anterior and posterior dislocation, respectively. In conclusion, the safe zone alone is not sufficient to prevent dislocation following THA; acetabular component position is influenced by multiple factors, and although standard anteversion and inclination angles have been defined, component positioning should be individually adjusted intraoperatively according to hip stability [38]. This may be related to spinopelvic mobility, head–neck length, and anatomical variations. In previous studies, it has been reported that accurate positioning of the acetabular component can be improved with the assistance of robotic surgery [39,40].

In surgical stability, implant design and surgical experience play a critical role as well as anatomical restoration [39]. Indeed, the literature has widely reported that head sizes of 28 mm and smaller are associated with increased risk, whereas 32–36 mm heads reduce the risk by increasing range of motion [41,42,43,44,45,46,47,48]. In our study, the marked reduction in dislocation rates (1.6%) observed after 2010, when the routine use of large-diameter (32–36 mm) femoral heads was introduced, is fully consistent with these findings. No dislocations were observed in the 32 mm group, whereas among the four dislocations in the 36 mm group, two were associated with abnormal early postoperative trauma (squatting, falls), and the remaining two were related to aseptic loosening in specific metal-on-metal prostheses with poor long-term outcomes. The ideal femoral head diameter has been reported to be 32–36 mm in the literature [42]. This diameter range has been reported to contribute to a greater range of motion without dislocation of the femoral head [40]. In many studies, femoral head diameters of 28 mm and smaller have been reported as a risk factor for dislocation, whereas femoral head diameters of 32 mm and larger have been shown to reduce the risk of dislocation [43,44,45,46,47]. Similarly, lipped liners have been reported to reduce dislocation rates by providing additional support in regions prone to instability [8]. In a study, 10° lipped liners were compared with 0° neutral liners, and the dislocation rate was reported to be significantly lower in cases using lipped liners [8]. Also, it has been suggested that lipped liners increase the distance required for the femoral head to dislocate from the acetabular insert, thereby potentially reducing the risk of dislocation through this mechanism [25,48]. It is known that implant quality plays an important role in the development of dislocations [7]. The use of implants that do not meet international standards, lack well-established long-term outcomes, have unproven efficacy, and are of low quality may lead to aseptic loosening [9]. In our study, a total of four dislocation cases associated with aseptic loosening were identified; two were related to the same prosthesis brand, and the other two were associated with metal-on-metal prostheses from the same manufacturer. All of these dislocations were observed to occur within the first 10 years. Despite the stability advantage conferred by large-diameter metal-on-metal bearing surfaces, several important biological complications have been reported with their long-term use. Cobalt and chromium ions, together with metal wear particles released from metal-on-metal articulations, may induce local adverse tissue reactions, osteolysis, pseudotumor formation, and ultimately aseptic loosening. Also, systemic exposure to metal ions is among the significant safety concerns associated with this group of implants. Long-term follow-up studies have shown that metal ion levels are elevated, particularly in large-diameter metal-on-metal total hip arthroplasties, and that this may be associated with clinical and radiographic failure [49]. Therefore, when evaluating the beneficial effects of larger femoral head diameters on hip stability, the biological safety and long-term performance of the bearing surface should also be taken into consideration.

In previous studies, surgical experience has been shown to reduce dislocation rates; the dislocation rate was reported to be 1.5% in surgeons performing more than 50 THAs per year, compared with 4.2% in those performing five or fewer procedures annually [50,51]. In addition, it has been reported that each additional 10 THAs performed annually reduces the surgeon-related dislocation rate by approximately 50% [52]. The highest dislocation rate (6.8%) was observed during 1996–2000, corresponding to the surgeon’s early experience with primary total hip arthroplasty. However, over the years, a gradual decline in dislocation rates has been observed. This may be related to increasing surgical experience. However, over the course of the study, changes occurred not only in surgical experience but also in surgical practice, including the adoption of techniques aimed at preserving the posterior capsule, the use of larger femoral head diameters, and advances in implant design. Hence, it is not possible to attribute the observed decrease solely to surgical experience. The reduction in dislocation rates is therefore more likely to reflect the combined effects of increasing surgical experience together with advances in surgical technique and implant-related factors. Due to the retrospective design of the study, the independent effects of these factors could not be separated from one another. In our study, although posterior capsular repair did not reach statistical significance in preventing dislocation, it showed a clinical trend toward reducing the risk of dislocation. However, despite reports of failure in some studies [27,53,54,55], the observed clinical protective trend suggests that this repair may act not merely as a biomechanical barrier but rather as a proprioceptive restraint that becomes tensioned during extreme movements, thereby limiting excessive motion.

In THA instability, the influence of patient-related demographic variables, in addition to surgical factors, remains controversial. When the age parameter was analyzed, some studies reported an increased risk of dislocation in patients over 70 years of age, whereas others have indicated a higher risk in those over 80 years of age [56]. This has been suggested to be associated with sarcopenia, the development of neurological disorders, an increased risk of falls, and spinopelvic mismatch secondary to reduced lumbosacral spinal mobility [56,57,58]. In contrast, some studies have reported an increased risk of dislocation in patients younger than 50 years, which has been attributed to higher activity levels and a greater tendency toward demanding movements in younger patients [57,58,59,60,61]. In our analyses, no statistically significant association was found between age and dislocation across all age groups (≤50, 51–65, 66–80, and >80 years). However, the highest dislocation rate was observed in the >80 years group (7.7%), whereas the lowest rate was observed in the 66–80 years group (1.9%).

Some studies have reported no direct association between gender and dislocation [15,62]. In contrast, some studies have reported that the incidence of dislocation is approximately twice as high in female patients compared with males; however, this difference is particularly pronounced in women younger than 55 years, while the rates become comparable between female and male patients over 75 years of age [2]. Our study found that the rate was significantly higher among male patients (8.0%) than among female patients (2.9%). This finding is thought to be directly associated with dislocation resulting from inadequate femoral offset secondary to insufficient restoration of the anatomical head–neck offset in male patients.

In our study, multiple dislocations were observed in one patient due to a neuromuscular disease developing after THA and in another patient due to abductor muscle weakness following lumbar disc herniation surgery. In both patients, a constrained liner was used, and no recurrent dislocation was observed during follow-up. In the literature, abductor muscle insufficiency and neuromuscular disorders have been reported to cause dislocation in 7.5–10.6% of cases [2,8]. In patients at high risk of dislocation, dual-mobility implants and constrained liners are preferred implant options to enhance stability. Dual-mobility systems reduce the risk of dislocation through increased range of motion and greater jump distance, whereas constrained liners may provide additional stability, particularly in patients with abductor insufficiency, neuromuscular disorders, and severe soft tissue deficiency. However, constrained liners have been reported to require careful patient selection due to increased mechanical stress and the risk of implant failure [63,64]. In our study, constrained liners were used in two high-risk patients with neuromuscular disorders and abductor insufficiency, and no recurrent dislocation was observed during follow-up. These findings support that the use of constrained liners in appropriately selected patients may be an effective option for preventing instability [63].

When the effects of secondary hip osteoarthritis etiologies on dislocation risk were analyzed, no statistically significant association was found between DDH and dislocation following THA. Recent studies have also reported no significant association between DDH and dislocation following THA [65]. In the literature, conditions such as AS, which involve spinal involvement and impair spinopelvic mobility, have been reported to increase the risk of dislocation [2,66,67]. However, the lack of a statistically significant association between AS and postoperative dislocation in our study may be attributed to the small number of patients with AS, regular follow-up and treatment by the rheumatology department, and the absence of advanced spinal involvement. In patients undergoing THA due to FAVN, the risk of dislocation has been reported to be 1.48 times higher compared with patients with primary osteoarthritis [6,68]. This has been suggested to be associated with the fact that patients with FAVN, compared with those with osteoarthritis, are generally younger, more frequently male, of larger body habitus, and have higher levels of physical activity [6,18,20,63]. In our study, no statistically significant association was found between FAVN and dislocation following THA. This may be attributed to the fact that 32–36 mm femoral heads were used in 22 of the 42 hips (52.4%) treated with THA due to FHAVN.

In our study, 72.7% of dislocations occurred more than three months after surgery. This rate differs from that reported in many studies indicating that dislocations mostly occur in the early stages. However, there is no universally accepted time-based definition distinguishing early and late dislocation. In the literature, different cutoff values, including three months [7,28], six months [4], and one year [29], have been used. Therefore, the reported rates of early and late dislocation may be influenced by the time-based definition used. The high rate of late dislocation observed in our study may be associated with the three-month classification threshold, as well as the duration of follow-up and the characteristics of the study population. Therefore, our findings should be interpreted within the context of our study cohort rather than being generalized to all primary total hip arthroplasties.

This study has some limitations. First, the retrospective design and single-center setting of the study limit the generalizability of the findings to broader populations. Furthermore, because the study covered an approximately 29-year period, substantial changes occurred during this time in surgical techniques, implant designs, femoral head diameters, bearing surfaces, and perioperative practices. In addition, the increase in the surgeon’s experience over time represents an important temporal confounding factor that makes it difficult to attribute the observed outcomes to a single factor. Additionally, the exclusion of patients with cognitive or mental disorders who were at increased risk of dislocation due to noncompliance may have introduced potential selection bias; therefore, our findings may only be generalizable to cognitively intact populations. The heterogeneity in follow-up durations among patients may affect the interpretation of the results, as it may lead to differences in the likelihood of detecting complications that develop in the late postoperative period. Similarly, the possibility of survival bias should be considered, as patients with available long-term follow-up data were included in the evaluation. In this study, each hip in patients who underwent bilateral THA was considered an independent observation, and the potential statistical dependence between the two hips of the same patient was not accounted for in the analyses. Furthermore, because the number of dislocation events was limited relative to the number of variables investigated, multivariable analysis could not be performed, and the independent effects of potential confounding variables could not be assessed. Consequently, the findings obtained in this study reflect associations between variables rather than causal relationships.

An important limitation of the study is that femoral offset deficiency and acetabular component malposition were not objectively assessed using standardized radiographic or computed tomography measurements. Similarly, the classification of dislocation causes was based on intraoperative clinical findings obtained during revision surgery and relied on the surgeon’s clinical assessment. Accordingly, this classification is observer-dependent and may be subject to classification bias arising from subjective assessment. Also, because spinopelvic parameters were not systematically evaluated using standardized radiographic methods, the potential effects of spinopelvic mobility and alignment on the development of dislocation could not be analyzed.

Finally, the low number of cases in some secondary etiology subgroups, such as AS and FAVN, increases the possibility of a type II error. Moreover, the limited number of dislocation events relative to the number of variables investigated may have reduced the statistical power and prevented the detection of some associations. Future larger-scale, multicenter, and prospective studies incorporating standardized radiographic assessments, spinopelvic analyses, and multivariable statistical methods are needed to validate these findings and enable more robust interpretation of the results.

## 5. Conclusions

Instability developing after primary THA is a multifactorial complication. In this study, femoral offset deficiency and acetabular component malposition were identified as the most frequently observed mechanical causes during revision surgery. Additionally, a significant association was observed between male sex and the development of dislocation, whereas the use of femoral heads measuring 32 mm or larger was associated with lower dislocation rates. However, given the retrospective design of the study, the changes in surgical techniques, implant technologies, and surgical experience over the approximately 29-year study period, as well as the inability to perform multivariable analysis, the independent effects of these factors on the development of dislocation cannot be definitively established.

These findings suggest that careful consideration of component positioning and restoration of the femoral offset as anatomically as possible may be important for achieving stability in primary total hip arthroplasty. However, the potential contributions of technologies such as robotic surgery and computer-assisted navigation to component placement accuracy and instability were not evaluated in this study, and this issue should be investigated in future prospective comparative studies. Similarly, the potential role of posterior capsular repair in reducing the risk of dislocation could not be definitively demonstrated in the present study, and this association requires confirmation in studies with larger sample sizes.

## Figures and Tables

**Table 1:** Demographic data.

	Mean ± SD	Median (Min:Max)	Q1	Q3
Age (year)	56.85 ± 11.95	57 (21:87)	49	66
Follow up (month)	79.4 ± 75.6	47(6:357)	16	127
			**Frequency**	**Percentage**
**Gender**				
Male			261	24.5
Female			805	75.5
**Hip osteoarthritis**				
Right			295	27.7
Left			310	29.1
Bilateral			461	43.2
**Hip osteoarthritis (Etiology)**				
Primary			735	68.9
Secondary			331	31.1
**Total hip arthroplasty**				
Right			359	40.0
Left			369	41.1
Bilateral			169	18.9
**Type of Prosthesis**				
Cemented			4	0.4
Cementless			957	89.8
Hybrid			105	9.8
**Type of Anesthesia**				
Spinal anesthesia			633	59.4
Spinal + Epidural Anesthesia			292	27.4
General anesthesia			141	13.2
**Repair status of posterior capsule**				
No repair of the posterior capsule + repair of external rotators			513	48.1
Repair of the posterior capsule and external rotators			553	51.9
**Head sizes used**				
22 mm			25	2.3
26 mm			2	0.2
28 mm			788	73.9
32 mm			109	10.2
36 mm			142	13.3
**Angles of the liners used**				
0°			17	1.6
10°			923	86.6
15°			63	5.9
20°			63	5.9

SD, standard deviation; min, minimum; max, maximum; Q1, 25th percentile; Q3, 75th percentile.

**Table 2:** Direction, timing, and annual incidence of dislocations following THA.

	Frequency	Percentage
**Dislocation status**		
No	1022	95.9
Yes	44	4.1
**Direction of Dislocation**		
Posterior	43	97.7
Anterior	1	2.3
**Time of Dislocation**		
Early	12	27.3
Late	32	72.7
**For 28 mm heads prior to 2010**		
No repair of the posterior capsule + repair of external rotators	501	68.2
Repair of the posterior capsule and external rotators	234	31.8
	**Number of Cases n (%)**	**Total Number of Dislocations n (%)**
**Distribution of dislocations by year**		
1996–2000	118 (18.2)	8 (6.8)
2001–2005	314 (43.2)	19 (6.1)
2006–2009	319 (27.3)	12 (3.8)
>2010	315 (11.4)	5 (1.6)

**Table 3:** Causes of dislocation following THA and applied treatment strategies.

Reason for Dislocation	n	%	Treatment Applied
**Acetabular component malposition**	14	31.82	
Cup anteversion error	4	9.09	Cup anteversion corrected
Cup inclination error	2	4.55	Cup inclination corrected
Cup anteversion + inclination error	2	4.55	Cup anteversion and inclination corrected
Cup anteversion + inclination + FO error	1	2.27	Cup anteversion and inclination corrected, head length increased
Cup anteversion + FO error	2	4.55	Cup anteversion corrected, head length increased
Cup inclination + FO error	2	4.55	Cup inclination corrected, head length increased
Cup inclination error + aseptic loosening	1	2.27	Cup revision was performed
**FO Error**	18	40.91	Head length and/or head diameter was increased depending on the case
**Other Reasons**	12	27.27	
Trauma, deep sitting, and mismatch	6	13.64	Closed reduction performed
Neuromuscular disease and postoperative paresis following lumbar surgery	2	4.55	Constrained liner was used.
Aseptic loosening	4	9.09	Cup revision was performed
**Total**	44	100	

FO, femoral offset.

**Table 4:** The relationship between dislocation following THA and clinical, demographic, and surgical parameters.

Variables	Dislocation Status		χ^2^	*p*
	No	Yes		
Age (Year)	n (%)	n (%)		
≤50	296 (96.4)	11 (3.6)	7.302	0.055 ^a^
51–65	455 (94.4)	27 (5.6)		
66–80	259 (98.1)	5 (1.9)		
>81	12 (92.3)	1 (7.7)		
	**Mean ± SD**	**Mean ± SD**	**t**	***p***
**Age (year)**	56.80 ± 11.99	57.48 ± 10.43	−0.367	0.713 ^b^
	**No**	**Yes**	**χ^2^**	***p***
**Gender**	**n (%)**	**n (%)**		
Female	782 (97.1)	23 (2.9)		
Male	240 (92.0)	21 (8.0)		
**Femoral Head (mm)**				
22	23 (92.0)	2 (8.0)	14.219	0.006 ^a^
26	1 (50.0)	1 (50.0)		
28	751 (95.3)	37 (4.7)		
32	109 (100.0)	0 (0.0)		
36	138 (97.2)	4 (2.8)		
**Liner Angle**				
0	15 (88.2)	2 (11.8)	5.406	0.120 ^a^
10	888 (96.2)	35 (3.8)		
15	61 (96.8)	2 (3.2)		
20	58 (92.1)	5 (7.9)		
**For 28 mm heads prior to 2010**				
No repair of the posterior capsule + repair of external rotators	473 (94.4)	28 (5.6)	1.612	0.271
Repair of the posterior capsule and external rotators	226 (96.6)	8 (3.4)		
**Type of Anesthesia**				
Spinal anesthesia	604 (95.4)	29 (4.6)	1.019	0.618
Spinal + Epidural Anesthesia	281 (96.2)	11 (3.8)		
General anesthesia	137 (97.2)	4 (2.8)		
**Prosthesis Securing Method (Type of Prosthesis)**				
Cemented	4 (100.0)	0 (0.0)	1.302	0.522 ^a^
Cementless	919 (96.0)	38 (4.0)		
Hybrid	99 (94.3)	6 (5.7)		

^a^ Chi-square (χ^2^) and Fisher’s exact tests; ^b^ Independent samples *t*-test; SD, standard deviation.

**Table 5:** Cox regression analysis results regarding factors predicting hip prosthesis dislocation.

Variables	β	SE	Wald	df	*p*	HR	%95 CI
Age (year)	0.050	0.015	11.793	1	<0.001	1.052	1.022–1.082
Gender (Male vs. Female)	1.307	0.316	17.058	1	<0.001	3.694	1.987–6.869
Femoral head diameter	—	—	10.001	2	0.007	—	—
22–26 mm vs. 32–36 mm	2.649	0.855	9.599	1	0.002	14.145	2.647–75.595
28 mm vs. 32–36 mm	0.985	0.613	2.586	1	0.108	2.679	0.806–8.904
Insert Angle	—	—	7.893	3	0.048	—	—
0° vs. 20°	2.326	0.981	5.624	1	0.018	10.233	1.497–69.951
5° vs. 20°	0.013	0.523	0.001	1	0.980	1.013	0.364–2.823
10 vs. 20°	−0.313	0.870	0.130	1	0.719	0.731	0.133–4.024

CI, confidence interval; HR, hazard rate. Reference category: femoral head diameter of 32–36 mm. Model: χ^2^(7) = 37.76, *p* < 0.001. N = 1037 (events = 44, censored = 993).

**Table 6:** Evaluation of dislocation by the etiology of primary and secondary hip osteoarthritis.

Variables	Dislocation Status		*p*
	No	Yes	
Etiology	n (%)	n (%)	
Primary	704 (95.8)	31 (4.2)	0.870
Secondary	318 (96.1)	13 (3.9)	
**Secondary Causes**			
DDH	204 (97.6)	5 (2.4)	
Ankylosing spondylitis	14 (100.0)	0 (0.0)	
Femoral head avascular necrosis	41 (95.3)	2 (4.7)	
Rheum. arthritis	13 (86.7)	2 (13.3)	
Nonspecific arthritis	3 (75.0)	1 (25.0)	
Perthes sequelae	13 (100.0)	0 (0.0)	
Secondary post-traumatic	10 (83.3)	2 (16.7)	
Secondary to collum femoris fracture	4 (100.0)	0 (0.0)	
Coxa vara	7 (83.3)	0 (0.0)	
Endoprosthesis	4 (100.0)	0 (0.0)	
Otto pelvis (protrusio acetabuli)	2 (66.7)	1 (33.3)	
Femoral head–neck resection	1 (100.0)	0 (0.0)	
Post-osteotomy of the hip	2 (100.0)	0 (0.0)	

DDH, developmental dysplasia of the hip.

## Data Availability

The datasets used and/or analyzed during the current study are available from the corresponding author upon reasonable request.
